# Myeloid-specific CAMKK2 deficiency protects against diet-induced obesity and insulin resistance by rewiring metabolic gene expression and enhancing energy expenditure

**DOI:** 10.1016/j.molmet.2025.102250

**Published:** 2025-09-11

**Authors:** Andrea R. Ortiz, Kevin Nay, Brittany A. Stork, Adam M. Dean, Sean M. Hartig, Cristian Coarfa, Surafel Tegegne, Christopher RM. Asquith, Daniel E. Frigo, Brian York, Anthony R. Means, Mark A. Febbraio, John W. Scott

**Affiliations:** 1 Molecular and Cellular Biology, Baylor College of Medicine, Houston, TX, 77030, USA; 2 Drug Discovery Biology, Monash Institute of Pharmaceutical Sciences, Parkville, Victoria, 3052, Australia; 3 Department of Medicine, Division of Diabetes, Endocrinology, and Metabolism, Baylor College of Medicine, Houston, TX, 77030, USA; 4 School of Pharmacy, Faculty of Health Sciences, University of Eastern Finland, Kuopio, 70211, Finland; 5 Department of Cancer Systems Imaging, The University of Texas MD Anderson Cancer Center, Houston, TX, 77054, USA; 6 Department of Genitourinary Medical Oncology, The University of Texas MD Anderson Cancer Center, Houston, TX, 77054, USA; 7 Center for Nuclear Receptors and Cell Signaling, University of Houston, Houston, Texas, 77204, USA; 8 Department of Biology and Biochemistry, University of Houston, Houston, TX, 77204, USA; 9 St Vincent's Institute of Medical Research, Fitzroy, Victoria, 3065, Australia

**Keywords:** Kinase signaling, Inflammation, Insulin resistance, Glucose homeostasis, Liver steatosis

## Abstract

**Objective:**

Obesity is associated with chronic, low-grade inflammation in metabolic tissues such as liver, adipose tissue and skeletal muscle implicating insulin resistance and type 2 diabetes as inflammatory diseases. This inflammatory response involves the accumulation of pro-inflammatory macrophages in these metabolically relevant organs. The Ca^2+^-calmodulin-dependent protein kinase kinase-2 (CAMKK2) is a key regulator of cellular and systemic energy metabolism, and a coordinator of macrophage-mediated inflammatory responses. However, its role in obesity-associated metabolic dysfunction is not fully defined. The aim of this study was to determine the contribution of CAMKK2 to the regulation of inflammation and systemic metabolism during diet-induced obesity.

**Methods:**

Mice with myeloid-specific deletion of *Camkk2* were generated and challenged with a high-fat diet. Metabolic phenotyping, histological analyses, and transcriptomic profiling were used to assess whole-body metabolism, liver lipid accumulation, and gene expression in macrophages and adipose tissue.

**Results:**

Myeloid-specific *Camkk2* deficiency protected mice from high fat diet-induced obesity, insulin resistance and liver steatosis. These protective effects were associated with rewiring of metabolic and inflammatory gene expression in both macrophages and adipose tissue, along with enhanced whole-body energy expenditure.

**Conclusions:**

Our data establish CAMKK2 as an important regulator of macrophage function and putative therapeutic target for treating obesity and related metabolic disorders.

## Highlights

• Myeloid-specific CAMKK2 deficiency in mice prevents diet-induced fat accumulation, insulin resistance, and hepatosteatosis.
• CAMKK2 deficiency alters gene expression in macrophages, enhancing fat oxidation and driving an anti-inflammatory phenotype.
• CAMKK2 is an attractive therapeutic target for treating obesity and related metabolic disorders.

## 1 Introduction

Obesity is a major public health concern globally that results from a combination of sustained overnutrition and sedentary behavior, and is associated with the development of life-threatening comorbidities including type 2 diabetes, metabolic-dysfunction associated fatty liver disease (MAFLD), cardiovascular disease, and several cancers [[1], [2], [3]]. A characteristic feature of obesity is the presence of altered immune cell populations and chronic low-grade inflammation in metabolic organs such as white adipose tissue (WAT) and liver, which leads to a systemic pro-inflammatory state that associates with metabolic dysfunction [1,4]. Macrophages are thought to be a major driving force in obesity-induced inflammation [5]. The pathogenesis of obesity is associated with increased accumulation of pro-inflammatory macrophages in WAT and release of inflammatory cytokines and adipokines, which correlate with whole-body insulin resistance. In contrast, lean WAT contains an abundance of anti-inflammatory macrophages that are associated with insulin sensitivity [6]. Consequently, inhibiting pro-inflammatory macrophage infiltration and increasing the predominance of anti-inflammatory macrophages in WAT has been proposed as a therapeutic strategy to treat metabolic disorders caused by obesity-induced inflammation [4].

The cell signaling enzyme, Ca^2+^-calmodulin-dependent protein kinase kinase-2 (CAMKK2), is an important regulator of cellular and systemic metabolism that coordinates the function of key metabolic organs including adipose tissue and liver [7,8]. At the cellular level, nutrients and hormones activate CAMKK2 by increasing intracellular Ca^2+^ and promoting accumulation of the Ca^2+^-calmodulin complex. Germline deletion of *Camkk2* protects mice from high-fat diet-induced weight gain, insulin resistance, hepatic steatosis, and hepatocellular carcinoma [[9], [10], [11]]. Although hepatocytes constitute the dominant cell type in the liver, mice with hepatocyte-specific deletion of *Camkk2* fail to display the same resistance to hepatic lipid accumulation as their globally deleted counterparts [9,12]. These findings hint at the importance of other cell types in mediating the beneficial effects of *Camkk2* deletion on hepatic function and whole-body metabolism.

CAMKK2 is highly expressed in macrophages and regulates inflammatory responses but is largely absent in other immune cells of the myeloid lineage [13]. Since pro-inflammatory macrophages recruited to adipose tissue are thought to play a central role in obesity-induced metabolic dysfunction [5], we generated mice with myeloid-specific *Camkk2* deficiency to investigate the importance of CAMKK2 function within macrophages for obesity-induced insulin resistance.

## 2 Results

### 2.1 Myeloid *Camkk2* deficiency protects mice from high-fat diet-induced obesity via an increase in energy expenditure

**Figure 1:**
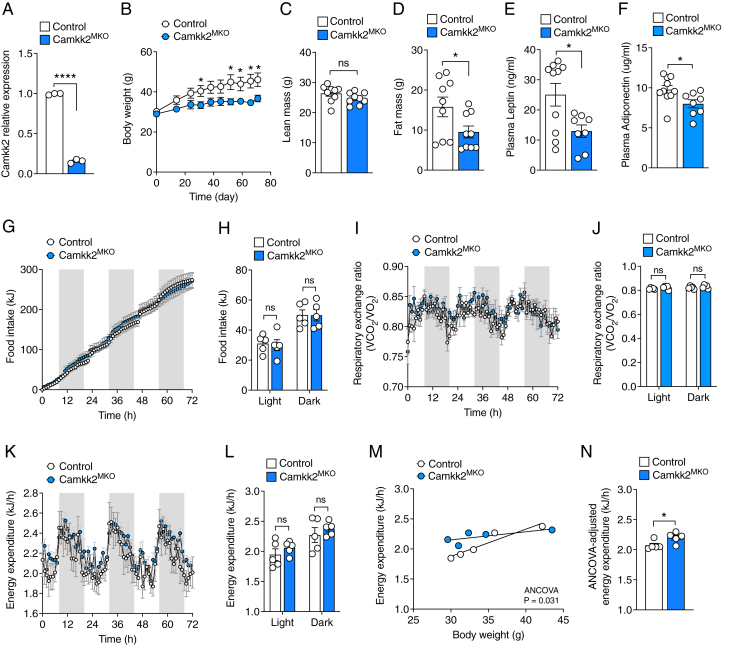
***Camkk2*^MKO^ mice are protected from diet-induced obesity**. qPCR analysis of *Camkk2* mRNA expression in bone marrow-derived macrophages (**A**), weekly body mass progression (**B**), lean mass (**C**), fat mass (**D**), plasma leptin (**E**), and plasma adiponectin levels (**F**) of *Camkk2*^MKO^ and control mice after 12 weeks on HFD. Cumulative food intake over a 72-hour period (**G**), average food intake within the light and dark cycle (**H**), respiratory exchange ratio over a 72-hour period (**I**), average respiratory exchange ratio within the light and dark cycle (**J**), energy expenditure over a 72-hour period (**K**), average energy expenditure within the light and dark cycle (**L**), regression plot comparing average daily energy expenditure measured over 72 h to body weight (**M**), and ANCOVA-adjusted energy expenditure (**N**), of *Camkk2*^MKO^ and control mice measured in metabolic cages over a 72 h period after 12 weeks on an HFD diet. For (A), (C), (D), (E), and (F): Unpaired t-test was used to analyze the data. For (B): Two-way repeated measures ANOVA was performed followed by Fisher's LSD post-hoc test; Control, *n* = 9, *Camkk2*^MKO^, *n* = 10. For (H), (J), (L) and (N): Two-way ANOVA was used followed by Sidak's post-hoc multiple comparisons test. Data are presented as means ± SEM. ∗*P* < 0.05, ∗∗∗∗*P* < 0.0001.

To investigate the role of CAMKK2 signaling in macrophages on whole-body metabolism, we conditionally deleted *Camkk2* in myeloid-lineage cells by generating mice homozygous for a floxed allele of *Camkk2* and homozygous for a transgene expressing Cre recombinase under the control of the *LysM* promoter (*Camkk2*^fl/fl^*LysM-Cre*^Tg/Tg^ termed *Camkk2*^MKO^ for simplicity). *Camkk2*^fl/fl^*LysM-Cre*^−/−^ littermates were used as controls. We confirmed efficient loss of *Camkk2* mRNA expression in bone marrow-derived macrophages (BMDMs) from *Camkk2*^MKO^ mice and control mice by qPCR (Figure 1A) and immunoblotting (Fig. S1A). *Camkk2* deletion had no impact on bone marrow progenitor cell numbers or their ability to differentiate into BMDMs (Fig. S1B–C).

We next studied the effect of myeloid *Camkk2* deficiency on whole-body metabolism under conditions of over-nutrition, by monitoring the body weight and adiposity of male *Camkk2*^MKO^ mice and age-matched controls fed a high-fat diet (HFD) over a period of 10 weeks. There was no significant difference in body weight prior to commencing the diet. However, *Camkk2*^MKO^ mice were resistant to weight gain during HFD feeding (Figure 1B). This effect of weight gain was not due to differences in lean mass (Figure 1C), but rather due to a significant reduction in total fat mass (Figure 1D). Consistent with this observation, *Camkk2*^MKO^ also displayed reduced plasma leptin levels, which is an indirect measure of body fat (Figure 1E). The *Camkk2*^MKO^ mice also displayed reduced adiponectin levels (Figure 1F). To investigate whether the protection from weight gain and adiposity in *Camkk2*^MKO^ mice were associated with changes to energy balance, we measured both food intake and whole-body energy expenditure. Food intake was not different when comparing *Camkk2*^MKO^ mice with controls (Figure 1G–H). Indirect calorimetry measurements revealed no differences in respiratory exchange ratio (Figure 1I–J), or total energy expenditure when expressed in absolute terms (Figure 1K–L). However, when we accounted for body weight differences using analysis of covariance (ANCOVA) as recommended by Speakman [14], ANCOVA adjusted energy expenditure was higher in *Camkk2*^MKO^ mice compared with control mice (Figure 1M−N). Taken together, these data suggest that *Camkk2*^MKO^ mice are resistant to diet-induced obesity due to enhanced energy expenditure rather than reduced food intake.

### 2.2 Myeloid *Camkk2* deficiency improves glycemic control and hepatosteatosis in mice fed a high-fat diet

**Figure 2:**
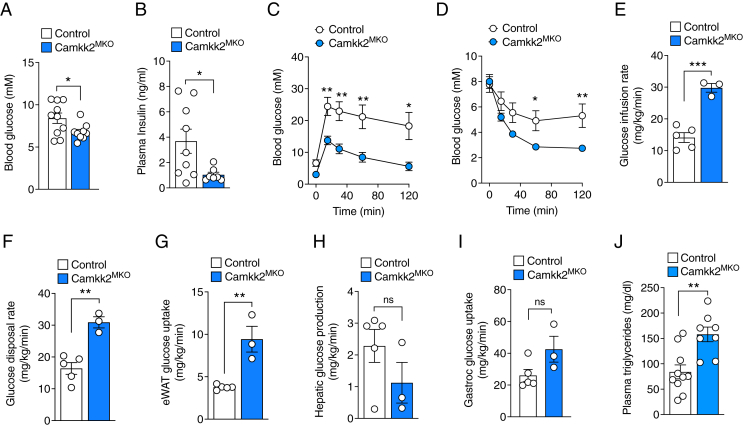
***Camkk2*^MKO^ mice display improved glycemic control on HFD.** Fasting blood glucose (**A**), plasma insulin (**B**), glucose tolerance test (**C**), insulin tolerance test (**D**), and hyperinsulinemic-euglycemic clamp data showing glucose infusion rate (**E**), glucose disposal rate (**F**), eWAT glucose uptake (**G**), hepatic glucose production post-clamp (**H**) and skeletal muscle glucose uptake (**I**), plasma triglyceride levels (**J**), in *Camkk2*^MKO^ and control mice after 12 weeks on HFD. For (A), (B), (E), (F), (G), (H), (I) and (J): Unpaired t-test was used to analyze the data. For (C) and (D): Two-way repeated measures ANOVA was performed followed by Fisher's LSD post-hoc test; Control, *n* = 10, *Camkk2*^MKO^, *n* = 9. Data are presented as means ± SEM. ∗*P* < 0.05, ∗∗*P* < 0.01, ∗∗∗*P* < 0.001.

Given the lean phenotype of *Camkk2*^MKO^ mice and the well-established link between obesity and poor glycemic control, we next assessed glucose homeostasis in *Camkk2*^MKO^ and littermate control mice fed an HFD. Both fasting glucose (Figure 2A) and insulin (Figure 2B) were lower in *Camkk2*^MKO^ mice compared with control mice. We next challenged the mice by measuring glucose and insulin tolerance. *Camkk2*^MKO^ displayed a marked improvement in both glucose (Figure 2C) and insulin (Figure 2D) tolerance compared with controls. To further investigate the improvements in glucose clearance and insulin responsiveness in *Camkk2*^MKO^ mice, we performed hyperinsulinemic-euglycemic clamp experiments, incorporating tritium (^3^H) and carbon-14 (^14^C) radioactive tracers to determine tissue-specific insulin sensitivities. *Camkk2*^MKO^ mice displayed a clear (p < 0.001) increase in glucose infusion rate (GIR) compared with control (Figure 2E). Furthermore, glucose disposal rate (GDR) was increased (Figure 2F) as was glucose uptake (p < 0.01) by epididymal (Figure 2G) WAT. While not statistically significant, hepatic glucose production (HGP) tended to be reduced (Figure 2H), while skeletal muscle (*gastrocnemius*) glucose uptake trended upwards (Figure 2I) in *Camkk2*^MKO^ mice compared with controls. Plasma triglyceride levels were also increased in *Camkk2*^MKO^ mice (Figure 2J), suggesting increased lipolysis. These findings demonstrate that mice lacking CAMKK2 expression in macrophages are protected from impairments in glucose homeostasis induced by a high-fat diet and that the liver, WAT and skeletal muscle undergo wholesale adaptations that improve energy balance.

**Figure 3:**
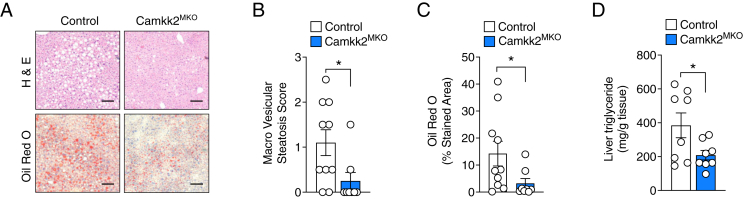
***Camkk2*^MKO^ mice are protected from HFD-induced hepatic steatosis.** Representative images of liver sections stained with H&E and Oil Red O (**A**), liver Oil Red O quantification (**B**), macro vesicular steatosis score (**C**), and liver triglyceride content (**D)**, of *Camkk2*^MKO^ and control mice after 12 weeks on HFD. Scale bars for (A): 100 μM. For (B), (C), and (D): Unpaired t-test was used to analyze the data. Data are presented as means ± SEM. ∗*P* < 0.05.

Given the marked decrease in fat mass and trend towards decreased HGP in the *Camkk2*^MKO^ mice compared with control, we next examined whether these mice were also protected from high-fat diet induced hepatosteatosis. Following HFD feeding, livers were harvested, paraffin embedded, sectioned and analyzed for hematoxylin and eosin (H&E) and Oil Red O staining (ORO) (Figure 3A). Macro vesicular steatosis (Figure 3B), ORO-stained area (Figure 3C), and liver triglycerides (Figure 3D) were all significantly reduced (p < 0.05) in *Camkk2*^MKO^ mice compared with control mice, suggesting that myeloid-specific loss of *Camkk2* protects against diet-induced lipid accumulation in the liver.

### 2.3 Myeloid-deficient *Camkk2* mice fed a HFD have reduced pro-inflammatory CD11c macrophages in eWAT and an anti-inflammatory transcriptional profile

**Figure 4:**
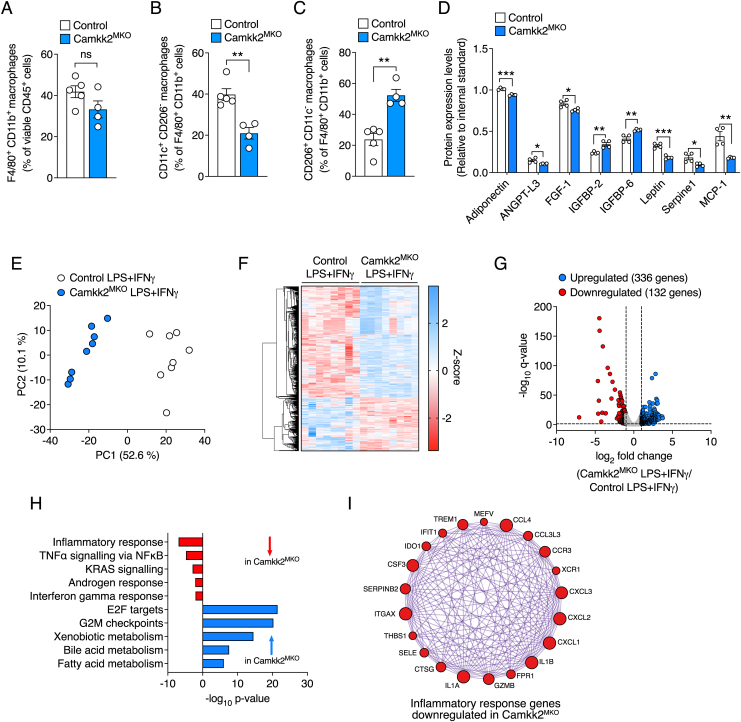
***Camkk2*^MKO^ mice on HFD have reduced CD11c^+^ inflammatory macrophages and an anti-inflammatory transcriptional profile.** Quantification of F4/80^+^CD11b^+^ macrophages as a percentage of viable CD45^+^ cells (**A**), CD11c^+^/CD206^-^ inflammatory macrophages as a percentage of F4/80^+^CD11b^+^ macrophages (**B**), CD206^+^/CD11c^−^ anti-inflammatory macrophages as a percentage of F4/80^+^CD11b^+^ macrophages (**C**), and adipokine expression (**D**) in eWAT extracted from *Camkk2*^MKO^ and control mice after 12 weeks on HFD. RNAseq data showing principal component analysis (**E**), heatmap and volcano plots of changes in gene expression (**F** and **G**), pathway enrichment analysis (**H**), and STRING protein–protein interaction analysis (**I**) of differentially expressed genes in naïve BMDMs treated with lipopolysaccharide (LPS) and interferon-γ (IFNγ) from *Camkk2*^MKO^ and control mice. For (A), (B), and (C): Unpaired t-test was used to analyze the data. For (D), two-way ANOVA was used followed by Sidak's post-hoc multiple comparisons test. Data are presented as means ± SEM. *P* < 0.05, ∗∗*P* < 0.01, ∗∗∗*P* < 0.001.

Epididymal white adipose tissue (eWAT) secretes an array of cytokines to regulate the metabolism of organs such as liver and skeletal muscle in HFD-induced obesity [15]. Accordingly, we next examined macrophage subtypes in eWAT in our mice by flow cytometry (Fig. S2A). Although consumption of a HFD failed to significantly alter the population of F4/80^+^ CD11b^+^ macrophages when comparing *Camkk2*^MKO^ mice with control mice (Figure 4A), myeloid-specific loss of *Camkk2* markedly decreased (p < 0.01) the percentage of CD11c^+^/CD206^-^ macrophages (Figure 4B), while conversely increased (p < 0.01) the percentage of CD206^+^/CD11c^−^ macrophages in eWAT tissue (Figure 4C). We next examined an array of chemokines and adipokines within eWAT. Monocyte chemoattractant protein-1 (MCP-1), a key chemokine that regulates migration and infiltration of macrophages into tissues, was markedly reduced (p < 0.01) in eWAT from *Camkk2*^MKO^ mice (Figure 4D). Adiponectin, Angiopoietin-like protein 3 (ANGPT-L3), Fibroblast growth factor-1 (FGF-1), Leptin and Serpine-1 were also reduced (p < 0.05), while Insulin-Like Growth Factor Binding Proteins-2 and -6 (IGFBP-2, IGFBP-6) were increased (p < 0.01) in *Camkk2*^MKO^ mice (Figure 4D).

Given the shift towards increased levels of CD206 macrophages and downregulation of MCP-1 expression in eWAT, we next examined whether *Camkk2* deficient macrophages more broadly exhibited anti-inflammatory gene transcriptional profiles. Accordingly, we obtained BMDMs from *Camkk2*^MKO^ and control mice, treated them with lipopolysaccharide (LPS) and interferon-γ (IFNγ) and performed RNAseq experiments. LPS/IFNγ treatment induced a rapid increase in Camkk2 expression in BMDMs from control mice, but not from *Camkk2*^MKO^ mice (Fig. S2B). Principal component analysis (Figure 4E), and fold changes in gene expression via heatmap (Figure 4F) and volcano (Figure 4G) plots are shown. Pathway enrichment analyses revealed that processes associated with inflammation and oncogenesis such as inflammatory response, TNF signaling, interferon gamma response, and KRAS signaling, were all downregulated in *Camkk2*^MKO^ relative to control mice, while metabolic pathways associated with fatty acid metabolism, xenobiotic metabolism and bile acid metabolism were all upregulated in *Camkk2*^MKO^ mice relative to controls (Figure 4H). Using the STRING database to collect, score and integrate protein–protein interactions, a cluster of key inflammatory response genes were found to be uniformly down-regulated in BMDMs obtained from *Camkk2*^MKO^ treated with LPS and IFNγ, relative to control BMDMs (Figure 4I). These data demonstrate that loss of CAMKK2 from BMDMs reprograms these cells to an inflammatory resistant state.

### 2.4 Myeloid-deficient *Camkk2* mice fed a HFD display a beiging transcriptional profile in both visceral and subcutaneous WAT

**Figure 5:**
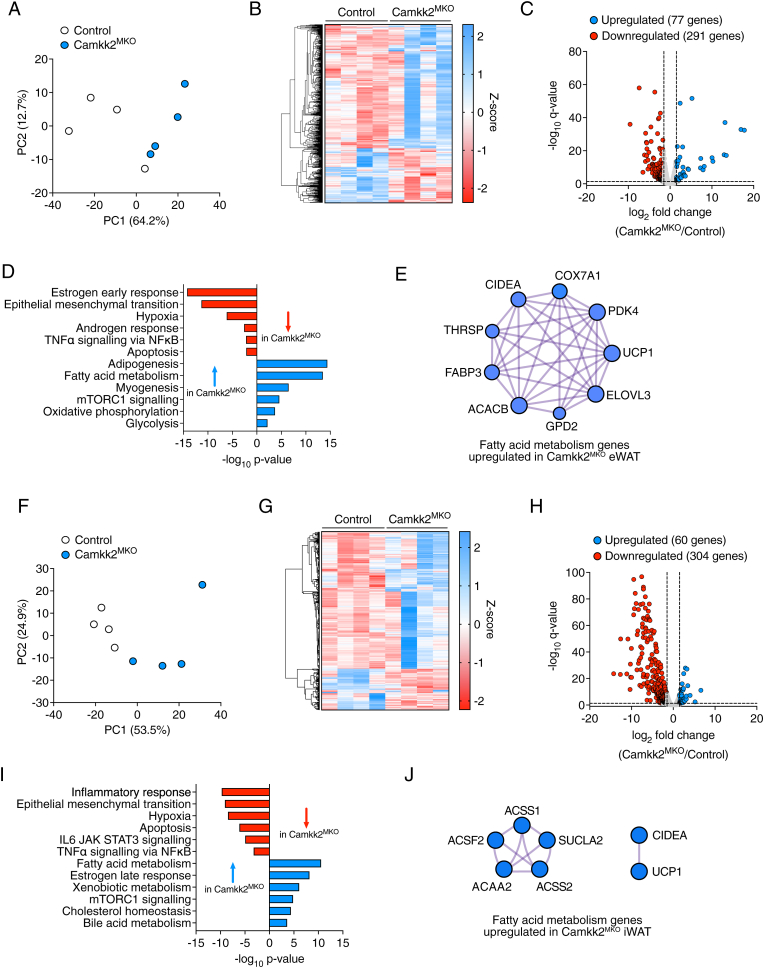
***Camkk2*^MKO^ mice fed a HFD display a beiging transcriptional profile in white adipose tissue.** RNAseq data showing principal component analysis (**A**), heatmap and volcano plots of changes in gene expression (**B** and **C**), pathway enrichment analysis (**D**), and STRING protein–protein interaction analysis (**E**) of differentially expressed genes in eWAT from *Camkk2*^MKO^ and control mice after 12 weeks on HFD. RNAseq data showing principal component analysis (**F**), heatmap and volcano plots of changes in gene expression (**G** and **H**), pathway enrichment analysis (**I**), and STRING protein–protein interaction analysis (**J**) of differentially expressed genes in iWAT from *Camkk2*^MKO^ and control mice after 12 weeks on HFD.

Given the data we had generated to this point, we next performed RNAseq experiments on eWAT samples obtained from *Camkk2*^MKO^ and control mice fed an HFD. Principal component analysis (Figure 5A), and fold changes in gene expression via heatmap (Figure 5B) and volcano (Figure 5C) plots are shown. Importantly, pathway enrichment analyses revealed that processes associated with fibrosis and inflammation such as epithelial to mesenchymal transition, hypoxia and TNF signaling were all downregulated in *Camkk2*^MKO^ mice relative to control mice, while pathways associated with increased energy utilization such as fatty acid metabolism, oxidative phosphorylation and glycolysis were all upregulated in *Camkk2*^MKO^ mice (Figure 5D). STRING analysis indicated protein–protein interactions of key factors associated with fatty acid metabolism and beiging of WAT such as Uncoupling Protein-1 (UCP1), Cell Death Activator-A (CIDEA) and Very Long Chain Fatty Acid Elongase-3 (ELOVL3) enriched in *Camkk2*^MKO^ eWAT (Figure 5E). We also performed RNAseq analysis on subcutaneous inguinal WAT (iWAT) from *Camkk2*^MKO^ and control mice fed a HFD, and observed a similar upregulation of proteins involved fatty acid metabolism and beiging relative to control mice (Figure 5F–J). Taken together, these data indicate that myeloid-specific loss of CAMKK2 expression decreases adiposity in HFD-fed mice by transcriptionally regulating pathways that increase fatty acid oxidation in both visceral and subcutaneous WAT.

### 2.5 Macrophages lacking *Camkk2* display increased rates of fatty acid oxidation

**Figure 6:**
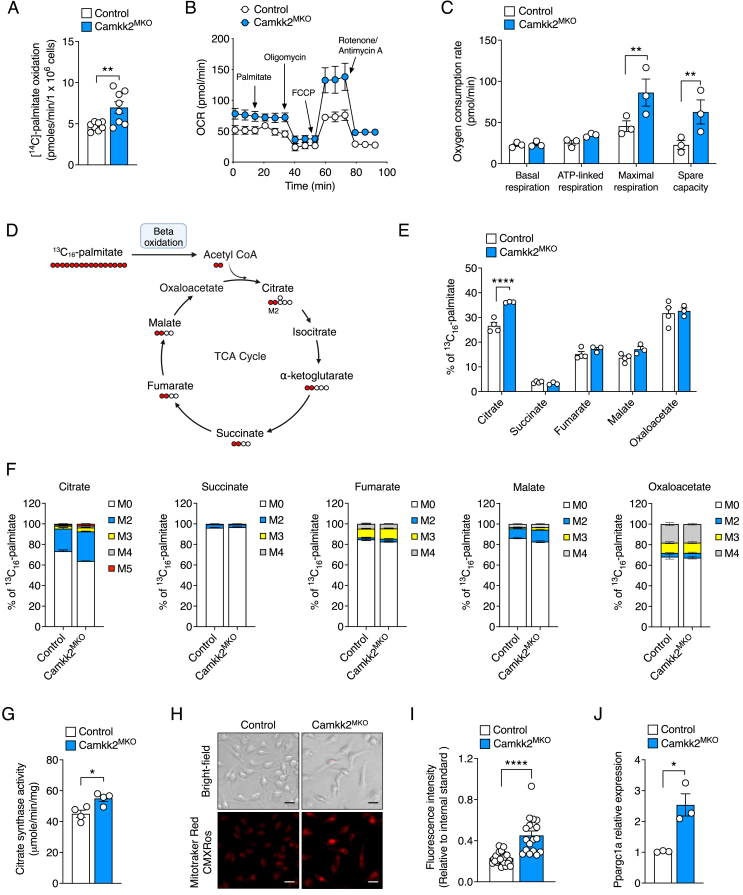
**Macrophages lacking CAMKK2 display increased rates of fatty acid oxidation.** Palmitate oxidation (**A**), oxygen consumption rate (**B**), basal respiration, ATP-linked respiration, maximal respiration, and spare capacity (**C**), schematic explaining metabolic tracing with ^13^C palmitate (**D**), labeling pattern of ^13^C palmitate into TCA metabolites (**E** and **F**), citrate synthase assay (**G**), mitochondrial staining with Mitotracker Red CMXRos (**H** and **I**), and qPCR analysis of *Ppargc1a* gene expression (**J**) in naïve BMDMs from *Camkk2*^MKO^ and control mice. Scale bars for (H): 10 μM. For (A), (G), (I) and (J): Unpaired t-test was used to analyze the data. For (C) and (E), two-way ANOVA was used followed by Sidak's post-hoc multiple comparisons test. Data are presented as means ± SEM. *P* < 0.05, ∗∗*P* < 0.01, ∗∗∗*P* < 0.0001.

Given the transcriptional profile of BMDMs and eWAT obtained from *Camkk2*^MKO^ relative to control mice described above, we next performed functional analyses in these macrophages. Naïve BMDMs obtained from *Camkk2*^MKO^ mice displayed higher (p < 0.01) palmitate oxidation relative to control BMDMs (Figure 6A). We next performed Seahorse assays to determine the oxygen consumption rates (OCR) of the BMDMs. When treated with 200 μM palmitate, both maximal respiration and spare capacity were increased (p < 0.01) in *Camkk2*^MKO^ relative to control BMDMs (Figure 6B–C). These data indicated a switch in the fuel preferences and ability of BMDMs from *Camkk2*^MKO^ mice to process select substrates for ATP production. Accordingly, we next examined whether deletion of *Camkk2* in BMDMs altered the function of the tricarboxylic acid (TCA) cycle. Entry into the TCA cycle typically begins with conversion of more complex metabolites into acetyl-CoA from glycolytic byproducts like pyruvate, or through the oxidation of lipids and glutamine sources. It is the irreversible catabolism of acetyl-CoA from these avenues that replenishes the TCA cycle intermediates, and eventually leads to the generation of 12 ATP molecules per metabolite. As we observed an increase in fatty acid oxidation of our *Camkk2* deficient macrophages (Figure 6A–C), we hypothesized that *Camkk2*^MKO^ BMDMs produce elevated amounts of acetyl-CoA and must possess a more active and functional TCA cycle. To test this hypothesis, we performed stable isotope ^13^C_16_-palmitate tracing in BMDMs isolated from *Camkk2*^MKO^ and control mice (Figure 6D). As predicted, our data showed increased TCA cycle flux from fatty acid precursors when *Camkk2* was ablated, indicated by an increase in the percentage of labeled citrate (Figure 6E–F) and to a lesser extent malate (Figure 6F). As citrate was the major TCA cycle intermediate that was affected by deletion of *Camkk2*, we next performed a maximal citrate synthase activity assay on the BMDMs. Citrate synthase catalyzes the first step of the TCA cycle, initiating the production of citrate from oxaloacetate and acetyl-CoA. It has been used as a marker for intact and functionally active mitochondria [16]. Citrate synthase maximal activity in BMDMs derived from *Camkk2*^MKO^ mice was significantly higher (p < 0.05) relative to BMDMs from control mice (Figure 6G). We paired these findings with immunofluorescent imaging of mitochondria using a MitoTracker dye and discovered that the absence of *Camkk2* in BMDMs resulted in less punctate and more mitochondria (Figure 6H–I). Consistent with these findings, we also found higher expression of *Ppargc1a* mRNA, a master coregulator of mitochondrial biogenesis and function, in BMDMs derived from *Camkk2*^MKO^ mice compared with controls (Figure 6J). Together, these functional assays demonstrate that BMDMs deficient in *Camkk2* have a remodelled state where oxidative metabolism is enhanced.

## 3 Discussion

The ability of macrophages to appropriately respond to inflammatory stimuli is inherently dependent on their capacity to reprogram their metabolic needs to match their required function. Herein, we identified a calcium and fatty acid responsive kinase, CAMKK2, as having direct control of regulating immune cell and whole-body metabolism. Under conditions of dietary stress or in cases of infection, reliance on aerobic glycolysis rather than oxidative phosphorylation for energy production predominates in pro-inflammatory macrophages because of a defunct and/or impaired TCA cycle [17,18]. This effect is, in part, mediated by the restructuring and fracturing of mitochondria that takes place during the polarization process. Without the capacity to further oxidize glycolytic byproducts in the mitochondria for ATP generation, pyruvate is instead utilized to generate certain TCA intermediates important for mediating inflammation or shunted towards lactic acid production [19,20]. Likewise, fatty acid oxidation in inflammatory macrophages is also downregulated due to the shift away from mitochondrial OXPHOS pathways for ATP generation [21]. Because distinct metabolic profiles are linked to macrophage inflammatory states, we sought to determine whether loss of CAMKK2 shifted this metabolic phenotype using stable isotope tracing. Our studies highlighted that BMDMs from *Camkk2*^MKO^ mice showed improved TCA cycle flux from lipid precursors, likely a direct result from the elevated fatty acid oxidation and acetyl-CoA generation we observed. Consequently, we demonstrate that myeloid-specific *Camkk2* deficient mice are protected from high fat diet-induced whole-body obesity, insulin resistance and hepatosteatosis.

Our data suggest that myeloid-specific deletion of *Camkk2* remodels adipose tissue gene expression toward a more metabolically favorable, anti-inflammatory state. In eWAT, loss of *Camkk2* in myeloid cells altered the pool of CD11c and CD206 macrophages, which was associated with a reduction in the expression of the chemoattractant MCP-1 and several adipokines. Transcriptomic profiling of *Camkk2*-deficient bone marrow-derived macrophages confirmed broad suppression of inflammatory gene expression and upregulation of genes involved in fatty acid oxidation, indicating that loss of CAMKK2 expression intrinsically reprograms macrophages toward an anti-inflammatory, metabolically active phenotype. Consistent with this cell-autonomous shift, transcriptomic profiling of eWAT and iWAT from *Camkk2*^MKO^ mice fed a high-fat diet uncovered downregulation of fibrosis and hypoxia-associated gene signatures and a coordinated upregulation of genes involved in fatty-acid oxidation, oxidative phosphorylation, and thermogenesis. Together, these findings identify CAMKK2 as a key metabolic rheostat in macrophages and demonstrate that CAMKK2 inhibition suppresses inflammatory gene expression, enhances lipid metabolism and energy expenditure, and thereby limits fat accumulation under high-fat diet conditions.

CAMKK2 and liver kinase-B1 (LKB1) are upstream activators of AMP-activated protein kinase (AMPK), a key enzyme involved in cellular energy sensing and maintenance of whole-body metabolic balance. One question that arises from our study is what role, if any, does AMPK play in mediating the phenotype we observed in *Camkk2*^MKO^ mice? The function of AMPK in macrophages—particularly in regard to the regulation of inflammation and whole-body energy metabolism—remains unclear, as findings from various genetic models have been inconsistent. In line with our results, mice with macrophage-specific deletion of the AMPKα1 subunit (*Prkaa1*) were found to be protected against high-fat diet-induced obesity and insulin resistance [22]. In contrast, other studies have shown that transplanting bone marrow from AMPKβ1-deficient mice into wild-type recipients fed a high-fat diet worsened adipose tissue inflammation and insulin resistance [23]. Additionally, macrophages lacking the AMPKβ1 subunit displayed decreased fatty acid oxidation and elevated inflammatory markers [23], opposite to what we observed in *Camkk2*-deficient macrophages. LKB1 has also been demonstrated to play a key role in modulating inflammation in macrophages and other cell types of myeloid lineage. Selective deletion of LKB1 in macrophages has been shown to increase inflammatory gene expression in response to LPS stimulation [24]. This response is the opposite of what we observed in *Camkk2*-deficient macrophages, suggesting that LKB1 is unlikely to serve a compensatory role when CAMKK2 is absent. Beyond macrophages, mice with LKB1-deficient dendritic cells develop more severe high-fat diet-induced hepatic steatosis, along with impaired glucose homeostasis and increased insulin resistance [25]. These metabolic disturbances are associated with disrupted signaling through AMPKα1 complexes and salt-inducible kinases (SIKs). Together, these findings indicate that LKB1 in myeloid cells plays a protective role in maintaining metabolic balance under high-fat diet conditions, whereas CAMKK2 appears to promote metabolic dysfunction in this context.

A limitation of our study is the basic phenotyping of eWAT macrophages using the commonly employed markers CD11c and CD206 to distinguish between pro- and anti-inflammatory populations [26]. Recent studies have highlighted the complexity and diversity of adipose tissue macrophages, identifying at least three distinct populations that share similar expression of canonical markers such as F4/80 and CD11b, but vary in CD11c expression [[27], [28], [29], [30]]. Notably, monocyte-derived CD11c macrophages can also express CD206, representing a hybrid phenotype observed in obese mouse eWAT [31]. Consequently, studies relying on CD11c and CD206 to define or isolate macrophage subsets may inadvertently capture mixed populations. Moreover, the functional roles of CD11c and CD206 that historically define macrophage subtypes remain controversial and poorly defined [[32], [33], [34]]. Other macrophage populations, including vascular and lipid-associated subtypes, have also been reported to play important roles in metabolic health [30,35,36]. Therefore, a more comprehensive characterization of eWAT macrophages in *Camkk2*^MKO^ mice would be valuable in future studies. A second, albeit minor, limitation of our study is that *LysMCre*-driven conditional gene deletion affects not only macrophages but also partially targets dendritic cells and neutrophils. Since neutrophils do not express CAMKK2 and dendritic cells are far less abundant than macrophages, the effects of *LysMCre*-mediated *Camkk2* deletion are most likely attributable to macrophages [13,37]. A third limitation of this study is that, although our *in vitro* data clearly demonstrate that CAMKK2 deficiency reprograms macrophage metabolism toward lipid utilization, it remains unresolved whether this metabolic shift results in increased lipid uptake *in vivo*. Addressing this question will require future *in vivo* metabolic tracing studies to quantify lipid uptake by CAMKK2-deficient macrophages and to elucidate the specific sources of lipids within the tissue microenvironment.

Although we focused on obesity-induced metabolic dysfunction in this study, it should be noted that macrophage polarity and, importantly, metabolic reprograming is also implicated in infectious and/or inflammatory diseases [38], atherosclerosis [4] and cancers [39]. Management of inflammation within macrophages can be key in the prevention and treatment of these diseases. We suggest, therefore, that CAMKK2 could be a promising therapeutic target, specifically in macrophages, for several diseases. Interestingly, CAMKK2 may regulate macrophage inflammation in a tissue or environment-specific manner. Prior work demonstrated that myeloid-specific deletion of *Camkk2* impaired syngeneic tumor growth in preclinical models of breast cancer [40]. However, in this context, CAMKK2 promoted an immune-suppressive tumor microenvironment that could be reversed in germline *Camkk2* knockout or STO-609-treated mice. Whether the differences in CAMKK2-regulated immune functions are due to differences between conditional versus systemic CAMKK2 ablation and/or tissue-influenced programs is currently not known. Clearly, the challenge would be to target drugs and drug carriers specifically to distinct macrophage populations. To that end, strategies are being formulated to develop drug delivery systems designed to specifically target macrophages [41]. Unfortunately, to date, almost all CAMKK2 pharmacological studies have utilized the relatively non-specific inhibitor, STO-609, which is suboptimal because it inhibits multiple protein kinases beyond CAMKK2, has micromolar on-target inhibition, and poor pharmacokinetic properties [42]. In addition, STO-609 potently activates the aryl hydrocarbon receptor [43]. Recently, our group has developed a series of highly potent and selective CAMKK2 inhibitors as starting points for medicinal chemistry optimization [[44], [45], [46]]. Ligand-directed degraders of CAMKK2 have also been developed and shown to impair migratory capacity and metastatic potential of triple negative breast cancer cells [47]. Hence, the development of clinically relevant CAMKK2 inhibitors may fill a large unmet clinical need for new therapies for inflammatory diseases, including chronic co-morbid conditions of obesity.

In summary, we demonstrate that deletion of *Camkk2* within macrophages generates a metabolically beneficial phenotype in metabolic tissues, increasing their anti-inflammatory transcriptional profile and enhancing their capacity to use lipids as substrates. Consequently, mice with a myeloid specific deletion of *Camkk2* are protected from high fat diet-induced obesity, insulin resistance and hepatosteatosis, highlighting CAMKK2 as an attractive therapeutic target for the treatment of inflammatory and metabolic diseases.

## 4 Methods

### 4.1 Mouse husbandry

All mouse experiments were performed in accordance with the Animal Care Research Committee at Baylor College of Medicine. *Camkk2*^fl/fl^*LysM-Cre*^Tg/-^ mice were generated by crossing *Camkk2*^fl/fl^ mice with *LysM-Cre*^Tg/-^ mice. The *Camkk2*^fl/fl^*LysM-Cre*^Tg/-^; mice were then crossed together to generate *Camkk2*^fl/fl^*LysM-Cre*^−/−^ (Control) or *Camkk2*^fl/fl^*LysM-Cre*^Tg/Tg^ (*Camkk2*^MKO^) mice. All mice were bred and maintained on a pure C57BL6/J background. Mice were maintained in a temperature controlled (23 °C) facility with a 12 h light/dark cycle. Mice were fed either a normal chow or high fat (Bioserve; #F3282, Fat calories 60 %) mouse diet ad libitum with free access to food and water. All experiments were conducted using male mice due to their increased susceptibility to diet-induced obesity and metabolic dysfunction.

### 4.2 Histology

Fresh liver tissue was fixed in 4 % paraformaldehyde for 48 h and embedded in paraffin. Hematoxylin and eosin (H&E) staining was used to evaluate gross morphology, and Sirius Red was used to examine fibrosis. Frozen-block preparation was performed by embedding liver tissues in OCT compound. Oil Red O staining was used to visualize neutral lipid accumulation as previously described. All staining was carried out by the pathology core at Baylor College of Medicine.

### 4.3 Glucose and insulin tolerance tests

For glucose tolerance tests (GTT) with insulin measurement, mice were fasted for 16 h and administered a 50 % glucose solution via IP injection at 2 mg/kg. Blood samples were collected prior to, and after glucose injection at times 0, 15, 30, 60, and 120 min, respectively, via tail vein bleeding using a lancet. Blood glucose was measured based on the glucose hexokinase assay and plasma levels of insulin were determined by ELISA (Millipore). Mice were restrained repeatedly for less than a minute each time blood samples were collected. For insulin tolerance tests (ITT), mice were fasted for 4 h and administered a defined dose of insulin, 1 U/kg body weight (Humulin R) via IP injection. Blood glucose was measured at 0 (before) and after 15, 30, 60 and 120 min of insulin injection using a glucometer (Life Scan).

### 4.4 Hyperinsulinemic euglycemic clamp

Hyperinsulinemic euglycemic clamps were performed according to protocols previously described [48,49]. Specifically, *Camkk2*^MKO^ and littermate control mice were anesthetized, and a midline neck incision was made to expose the jugular vein. A microcannula was inserted into the jugular vein, threaded into the right atrium, and anchored at the venotomy site. Mice were allowed to recover for 4 days with ad libitum access to water and food. Following an overnight fast, the conscious mice received a primary infusion (10 μCi) and then a constant rate intravenous infusion (0.1 μCi/min) of chromatography-purified [3-^3^H]-glucose using a syringe infusion pump. For determination of basal glucose production, blood samples were collected after 50 and 60 min of labelled glucose infusion. After 60–90 min, mice were infused continuously for 2 h with human insulin (4 mU/kg/min). Simultaneously, 25 % glucose was infused using another infusion pump at a rate adjusted to maintain the blood glucose level at 100–140 mg/dl (euglycemia). Blood glucose concentration was measured every 9 min by a glucometer. Basal and hepatic glucose production rate after clamp, peripheral glucose disposal rate, and glucose infusion rate were then calculated. To estimate insulin-stimulated glucose uptake in individual tissues, [^14^C]-2-deoxyglucose (2DG) was administered as a bolus (10 μCi) at 45 min before the end of the clamps. At the end, mice were euthanized, and tissues were snap frozen using liquid nitrogen for tissue-specific glucose uptake. Glucose uptake in different tissues was calculated from plasma [^14^C]-2-deoxyglucose profile fitted with double exponential curve and tissue content of [^14^C]-2-deoxyglucose-6-phosphate.

### 4.5 RNAseq analysis

RNA samples were isolated from eWAT, iWAT and BMDMs extracted from *Camkk2*^MKO^ and control mice using the miRNeasy Mini Kit (Qiagen) per the manufacturer's instructions. For the BMDMs, samples were treated prior to RNA extraction with either vehicle (DMSO) or 100 ng/ml of LPS/IFNγ for 1 h at 37 °C. RNA samples (at least 1500 ng of total RNA for each eWAT sample and 1600 ng of total RNA for each BMDM sample) were processed by NovoGene for library prep, quality assurance, and sequenced with an average of 20 million paired end reads/sample. Gene Ontology analysis was performed using Metascape (https://metascape.org). Biological process relationships and disease perturbations were performed. Hallmark pathway analysis was used as the primary pathway category, and Mus musculus was set as background.

### 4.6 qPCR

Total mRNA was isolated from naïve BMDM from *Camkk2*^MKO^ and control mice with the Pure Link RNA Mini Kit (Invitrogen, #12183025), per the manufacturer's instructions. For cell lysates, total mRNA was isolated using the miRNeasy Mini Kit (Qiagen), per the manufacturer's instructions. Reverse transcription was carried out using 1.5–2 μg of RNA with Vilo Superscript Master Mix (Thermo Fisher) per the manufacturer's instructions. For gene expression analyses, cDNA samples were diluted 5-fold. Exactly 2.5 μl of diluted cDNA was combined with 18 μl of TaqMan Master Mix containing the appropriate primer pair as indicated by Roche Assay Design Center. β-actin was used as the internal control. All qPCR assays were run on a One Step-Plus Real Time PCR system (ThermoFisher).

### 4.7 Comprehensive lab animal monitoring systems (CLAMS)

Calorimetry (Columbus Instruments) was used for real time measuring of Respiratory Exchange Ratio (RER). *Camkk2*^MKO^ and control mice on a HFD for 12 weeks were fed ad libitum and acclimated to the metabolic cages for at least one week, after which food intake and energy expenditure was measured over 72 h period under a 12 h light/dark cycle. CalR (Version 1.3, https://calrapp.org) was chosen for the analysis of experiments using indirect calorimetry to measure physiological energy balance [50]. Data were collected for VO_2_ consumed and VCO_2_ released, and RER was calculated as VCO_2_/VO_2_.

### 4.8 Adipokine array

Adipose tissue from *Camkk2*^MKO^ and control mice placed on normal chow or high-fat diet for 14 weeks were isolated, processed and used in the Proteome Profiler Mouse Adipokine Array (R&D Systems) according to manufacturer's protocols.

### 4.9 Isolation of bone marrow derived macrophages (BMDM)

Mouse bone-marrow derived hematopoietic stem cells (HSCs) were isolated from male *Camkk2*^MKO^ and littermate control mice and cultured as previously reported with modifications [51]. Briefly, mice were anesthetized with isoflurane and then euthanized via rapid cervical dislocation. Following euthanasia, the rear legs were carefully degloved and stripped of all muscle and connective tissue. The hind legs were then cut at the hip and ankle joint, removed from the mouse and placed in 35 mm dishes containing 2 ml of ice-cold PBS (no Ca^2+^/Mg^2+^). The leg bones were then separated at the knee and cut on both ends of the femur and tibia using sterile surgical scissors. Using a 23G needle and 10 ml syringe, 5 ml of ice-cold complete Hanks balanced salt solution (+2 % HI-FBS and 20 mM HEPES) was used to flush out the bone marrow from each leg bone into a 10 cm petri dish until bones appeared opaque. An 18G needle was then used to break up bone marrow by repeatedly flushing solution against the dish surface. This step was repeated until no visible clumps remained. The resulting cells in media were then filtered into a 50 ml conical tube using a 70 μM filter and centrifuged at 400×*g* for 10 min at 4 °C. Once pelleted, cells were resuspended in 2 ml of 1x RBC lysis buffer (BD Pharmingen) and incubated at room temperature for 2 min. Following incubation, 8 ml of ice-cold complete Hank's balanced salt solution was added to each tube to neutralize the reaction. Cells were again centrifuged at 400×*g* for 10 min at 4 °C. Following incubation, supernatant was carefully removed, and cells were resuspended using a hand pipette in 20 ml of complete DMEM (high glucose) media supplemented with 20 mM HEPES, 1 mM sodium pyruvate, 1 % Pen/Strep and 10 % HI-FBS. Cells were counted using trypan blue and plated on 15 cm^2^ petri dishes at a density of ∼1.5 × 10^7^ cells per dish in 20 ml of complete DMEM. 10 ng/ml of macrophage-colony stimulating factor (M-CSF) was added to media prior to plating. This step was denoted Day 0. At Day 3, 15 ml of media was removed and replaced with 10 ml of fresh complete DMEM containing 10 ng/ml of M-CSF. At Day 5, 10 ml of media was again removed and replaced with an additional 10 ml of fresh DMEM containing 10 ng/ml of M-CSF. On Day 6, cells were harvested or used for characterization or analyses.

### 4.10 Immunoblotting

Immunoblot analyses were performed using SDS-PAGE. In brief, 20–25 μg of tissue protein lysate was loaded onto a 4–20 % gradient SDS-PAGE page gel, separated at 125 V for 90 min, transferred using the iBlot system for 5 min to nitrocellulose membranes, and blocked in 3 % BSA in Tris-buffered saline with 0.1 % Tween-20 (TBS-T). Primary antibodies (rabbit HRP conjugated β-actin, Cell Signaling #5125 S; mouse CaMKK2, BD Biosciences, 610545, RRID:AB_397902) were diluted in 3 % BSA in TBS-T at 1:1000, except for CAMKK2, which was used at a dilution of 1:400. Nitrocellulose membranes were incubated with primary antibodies overnight with rotation at 4 °C. Membranes were washed successively for 45 min in TBS-T and then incubated with appropriate secondary antibodies coupled to horseradish peroxidase (Digital anti- Mouse-HRP, R1005, 1:1000, Kindle Biosciences) for 1 h. Membranes were again washed in TBS-T, then treated with ECL reagents per the manufacturer's instructions (Thermo), and detected using a KwikQuant Imager (Kindle Biosciences).

### 4.11 Seahorse studies

BMDMs isolated from *Camkk2*^MKO^ and control mice were plated on XF96 well cell culture microplates (Agilent) at a density of 90,000 cells/well to form a consistent and confluent monolayer at the time of experimental measurements. Extracellular flux analysis was performed using the Seahorse XF Cell Mitochondrial Stress Test kit (Seahorse Bioscience P/N 103015-100) or Seahorse XF Glycolytic Rate Assay kit (Seahorse Bioscience P/N 102233-100). Experimental medium used was the Seahorse XF DMEM supplemented with 2.5 mM glucose, 1 mM sodium pyruvate, 2 mM glutamine, and 200 μM palmitate. For the Mito Stress Test, a final concentration of 2 μM oligomycin, 2 μM FCCP, and 0.5 μM rotenone/antimycin A was used. The assays were performed according to manufacturer's protocols using a Seahorse XF96 Analyzer.

### 4.12 Stable isotope tracing – ^13^C_16_-palmitate

BMDMs were isolated from *Camkk2*^MKO^ and control mice and allowed to differentiate as described above. On Day 6, cells were collected and plated on Primaria 15-cm tissue culture dishes (Corning) at a density of 5 million cells/plate and allowed to adhere at 37 °C for 6 h. Media was changed to starvation media (DMEM no glucose) and cells were incubated for 16 h at 37 °C. Dialyzed BSA (10 %) and Pen/Strep (1 %) were added to all plates. One % BSA Fraction V was added to all plates, along with either 150 μM of ^13^C_16_-palmitate (CLM3943; n = 3/genotype) or 150 μM of ^12^C-palmitate (Sigma P9767, n = 3/genotype). Metabolic extraction from cell pellets was performed as described previously [52]. Fatty acid ^13^C incorporation was measured by LC-MS using a Luna 3 μm Phenyl-Hexyl column (150 × 2 mm; (Phenomenex, Torrance, CA). The mobile phases A and B were 10 mM ammonium acetate (pH 8) and methanol. Gradient Flow: 0–8 min 40 % B, 8–13 min 50 % B, 13–23 min 67 %, 23–30 min 100%, and 30 min 40 %, followed by re-equilibration until the end of the gradient, approximately 37 min prior to the initial starting condition of 40 % B. Flow rate of the solvents used for analysis was 0.2 ml/min. The injection volume was 20 μL. The above mentioned sample volumes were injected and data were acquired via multiple reaction monitoring (MRM) in negative mode using a 6495 Triple Quadrupole mass spectrometry coupled to an Agilent UHPLC system (Agilent Technologies, Santa Clara, CA) through Agilent Mass Hunter Software [53]. The acquired data were analyzed, and integration of each peak was performed using Agilent Mass Hunter Quantitative Analysis software. Percentage of fatty acid ^13^C incorporation was calculated using Microsoft excel from peak area and represented as bar graphs.

### 4.13 Oil Red O staining

Oil Red O staining of cells was carried out using the Lipid (Oil Red O) Staining Kit (Sigma, #MAK194) according to the manufacturer's protocol. Retained Oil Red O was extracted using isopropanol and absorbance was measured at 492 nm.

### 4.14 β-oxidation assay

β-oxidation assays were performed as previously described [54]. Briefly, primary macrophages were plated in triplicate on 12-well plates at a density of 250,000 cells per well and allowed to adhere for 6 h. Media was then changed to serum starvation medium (DMEM-no glucose supplemented with 0.2 % BSA Fraction V, 2 mM sodium pyruvate, 2 % Pen/Strep, and 20 nM glucagon). After incubation at 37 °C for 12 h, the serum starvation medium was removed and 1 ml of pre-incubation medium (DMEM-no glucose supplemented with 25 mM HEPES, 1 % BSA Fraction V, 250 μM of sodium palmitate, and 20 nM glucagon) was added, and cells were incubated at 37 °C for 2 h. During the incubation period, the appropriate amount of 0.1 μCi/ml ^14^C-palmitate (0.5 μCi/well) was dried under air. Dried palmitate was resuspended in 0.1 N NaOH to a final concentration of 12.5 ml/μCi and incubated at 70 °C for 10 min. Three volumes of the pre-incubation media was then added to the palmitate and mixed via pipetting. Following the incubation period, 25 μl of diluted ^14^C-palmitate was spiked into each well and cells incubated for 90 min. During this incubation period, filter paper was added to the bottom of new 12 well plates (Filter Paper plate) to completely cover the lower surface of each well. Parafilm was then used to cover the top of the plate, and a 1.5 ml Eppendorf tube was used to seal edges around each well. Only the parafilm over well openings was carefully removed. 10 min before the end of the incubation period, 200–300 μl of 3 N NaOH was added to the filter paper of each well in the Filter Paper plate, ensuring complete absorption of liquid. Next, palmitate treated cells were snap frozen in liquid nitrogen and 200 μl of 70 % perchloric acid was added to each well. The previously made filter paper plate was then immediately placed on top, making sure to align wells correctly. Plates were quickly and carefully wrapped together with parafilm and left to rock at room temperature overnight for 16 h. The following day, filter papers were removed and placed into scintillation vials with 4 ml of liquid scintillation fluid. Vials were labelled, placed in a scintillation counter, and ^14^C counts were measured.

### 4.15 Citrate synthase assay and Mitotracker staining

BMDMs from *Camkk2*^MKO^ and control mice were isolated and differentiated *in vitro* as described above. On Day 7, cells were collected and plated in triplicate on 24 well plates at a density of 1 × 10^6^ cells per well and allowed to adhere for 2 h. Following incubation, cells were lysed and used in the Citrate synthase activity kit (Sigma-Aldrich, #MAK193) according to manufacturer's protocol. For Mitotracker staining, primary macrophages were isolated and plated in triplicate on 24 well plates at a density of 75,000 cells per well and allowed to adhere for 2 h. Following incubation, media was removed, and cells washed with 1 x PBS. The MitoTrakerTM Red CMXRos (ThermoFisher) was used to stain mitochondria according to the manufacturer's protocol, and visualized by fluorescence microscopy.

### 4.16 Flow cytometry

Stromal vascular fraction (SVF) cells from *Camkk2*^MKO^ and control mice were isolated from eWAT following digestion with collagenase type I (Worthington Biochemical Corporation). Cells were stained with Fc receptor blockade and antibodies to immune cell markers CD45 (Thermo Fisher #17-0451-82; RRID:AB_469392), F4/80 (Thermo Fisher #45-4801-82, RRID:AB_914345), CD11b (Thermo Fisher #14-0112-82, RRID:AB_467108), CD11c (Thermo Fisher #12-0114-82, RRID:AB_465552), and CD206 (Bio-Rad Laboratories #MCA2235F, RRID:AB_324594) or respective isotype controls. Data were collected with an LSRII cytometer (BD Biosciences) and analyzed using FlowJo. Gating was performed on viable CD45^+^ cells, from which total macrophages were identified as F4/80^+^ CD11b^+^ high. Macrophages subtypes were classified as CD11c^+^/CD206^–^ or CD206^+^/CD11c^–^ cells.

### 4.17 Statistical analyses

Statistical analyses were performed using Prism (Version 10.3.1; GraphPad). Comparisons between two groups were performed using unpaired, two-tailed Student's t-test. For comparisons of more than two genotype groups and effects, two-way ANOVA was performed followed by Sidak's post-hoc multiple comparisons test. Two-way repeated measures ANOVA were performed to compare two genotype groups analyzed with time, followed by Fisher's LSD post-hoc test. ANCOVA analysis was performed using body weight as a covariate using CalR (Version 1.3) [50]. Data were tested for normality and equal variance assumptions and log transformed prior to analysis when necessary. This approach reduced the total number of mice required per IACUC standards and all experiments adhered to ARRIVE guidelines.

## CRediT authorship contribution statement

**Andrea R. Ortiz:** Writing – original draft, Project administration, Methodology, Investigation, Data curation, Conceptualization. **Kevin Nay:** Writing – review & editing, Methodology, Investigation, Data curation. **Brittany A. Stork:** Writing – review & editing, Methodology, Investigation. **Adam M. Dean:** Writing – review & editing, Methodology. **Sean M. Hartig:** Writing – review & editing, Methodology. **Cristian Coarfa:** Writing – review & editing, Methodology. **Surafel Tegegne:** Writing – review & editing, Formal analysis. **Christopher RM. Asquith:** Writing – review & editing, Resources. **Daniel E. Frigo:** Writing – review & editing, Resources. **Brian York:** Writing – review & editing, Supervision, Resources, Funding acquisition, Conceptualization. **Anthony R. Means:** Writing – review & editing, Supervision, Resources, Funding acquisition, Conceptualization. **Mark A. Febbraio:** Writing – original draft, Supervision, Resources, Funding acquisition. **John W. Scott:** Writing – original draft, Supervision, Resources, Methodology, Funding acquisition.

## Funding

We acknowledge the joint participation by the Adrienne Helis Malvin Medical Research Foundation through its direct engagement in the continuous active conduct of medical research in conjunction with B.Y. at Baylor College of Medicine and the CAMKK2-PKM2 promotes HCC research program. C.R.M.A. is supported by an Academy of Finland PROFI6 grant. J.W.S. was supported by an NHMRC Ideas Grant (2001817) and an ARC Discovery Project (DP210102840). M.A.F. is supported by NHMRC Investigator Grants (1194141, 2040938). D.E.F. is supported by a CPRIT grant (RP240072).

## Declaration of competing interest

The authors declare the following financial interests/personal relationships which may be considered as potential competing interests: John W Scott reports financial support was provided by National Health and Medical Research Council. John W Scott reports financial support was provided by Australian Research Council. Brian York reports financial support was provided by Adrienne Helis Malvin Medical Research Foundation. Christopher Asquith reports financial support was provided by Academy of Finland. Mark A Febbraio reports financial support was provided by National Health and Medical Research Council. Daniel E Frigo reports financial support was provided by Cancer Prevention and Research Institute of Texas. Mark A Febbraio reports a relationship with Vitaleon Pharma that includes: consulting or advisory. Mark A Febbraio reports a relationship with Celesta Therapeutics that includes: equity or stocks. Daniel E Frigo reports a relationship with GTx Inc that includes: funding grants. Mark A Febbraio has patent #US-2020179363-A1 issued to Assignee. Daniel E Frigo has a familial relationship with Biocity Biopharmaceuticals, Hummingbird Bioscience, Bellicum Pharmaceuticals, Maia Biotechnology, Alms Therapeutics, Hinova Pharmaceuticals, and Barricade Therapeutics. If there are other authors, they declare that they have no known competing financial interests or personal relationships that could have appeared to influence the work reported in this paper.

## Data Availability

Data will be made available on request.

## Appendix A Supplementary data

Figure S1 *Camkk2*^MKO^ mice show loss of Camkk2 protein in BMDMs but no differences in bone marrow progenitor cell numbers or their ability to differentiate into BMDMs. Immunoblot image of Camkk2 expression in BMDMs from *Camkk2*^MKO^ and control mice (**A**). Bone marrow progenitor cell numbers (**B**) and BMDM cell numbers (**C**) from *Camkk2*^MKO^ and control mice. For (B) and (C): Unpaired t-test was used to analyze the data. Figure S1

Figure S2 Representative gating strategy to identify macrophages subsets in eWAT (**A**). Debris, doublets and non-viable cells were first excluded, after which total tissue leukocytes were identified as CD45^+^. The macrophage pool was identified as F4/80^+^CD11b^+^, from which CD11c^+^ and CD206^+^ macrophages were identified. (**B**) Immunoblot of time course of Camkk2 expression in BMDMs treated with lipopolysaccharide (LPS) and interferon-γ (IFNγ) from control and *Camkk2*^MKO^ mice. Figure S2
