# Supplementary figures and images for: Myeloid-specific CAMKK2 deficiency protects against diet-induced obesity and insulin resistance by rewiring metabolic gene expression and enhancing energy expenditure
Source: Mol Metab. 2025 Sep 11;101:102250. doi: 10.1016/j.molmet.2025.102250 (PMC12493246; doi:10.1016/j.molmet.2025.102250)

A

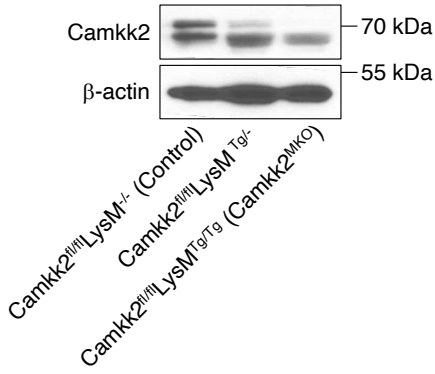

B

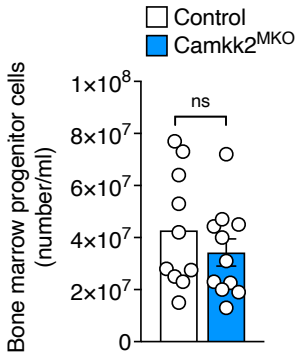

C

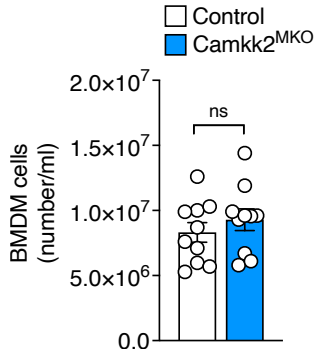

Supplement: Figure S1 — Camkk2MKO mice show loss of Camkk2 protein in BMDMs but no differences in bone marrow progenitor cell numbers or their ability to differentiate into BMDMs. Immunoblot image of Camkk2 expression in BMDMs from Camkk2MKO and control mice (A). Bone marrow progenitor cell numbers (B) and BMDM cell numbers (C) from Camkk2MKO and control mice. For (B) and (C): Unpaired t-test was used to analyze the data. [file mmc1.pdf]

A

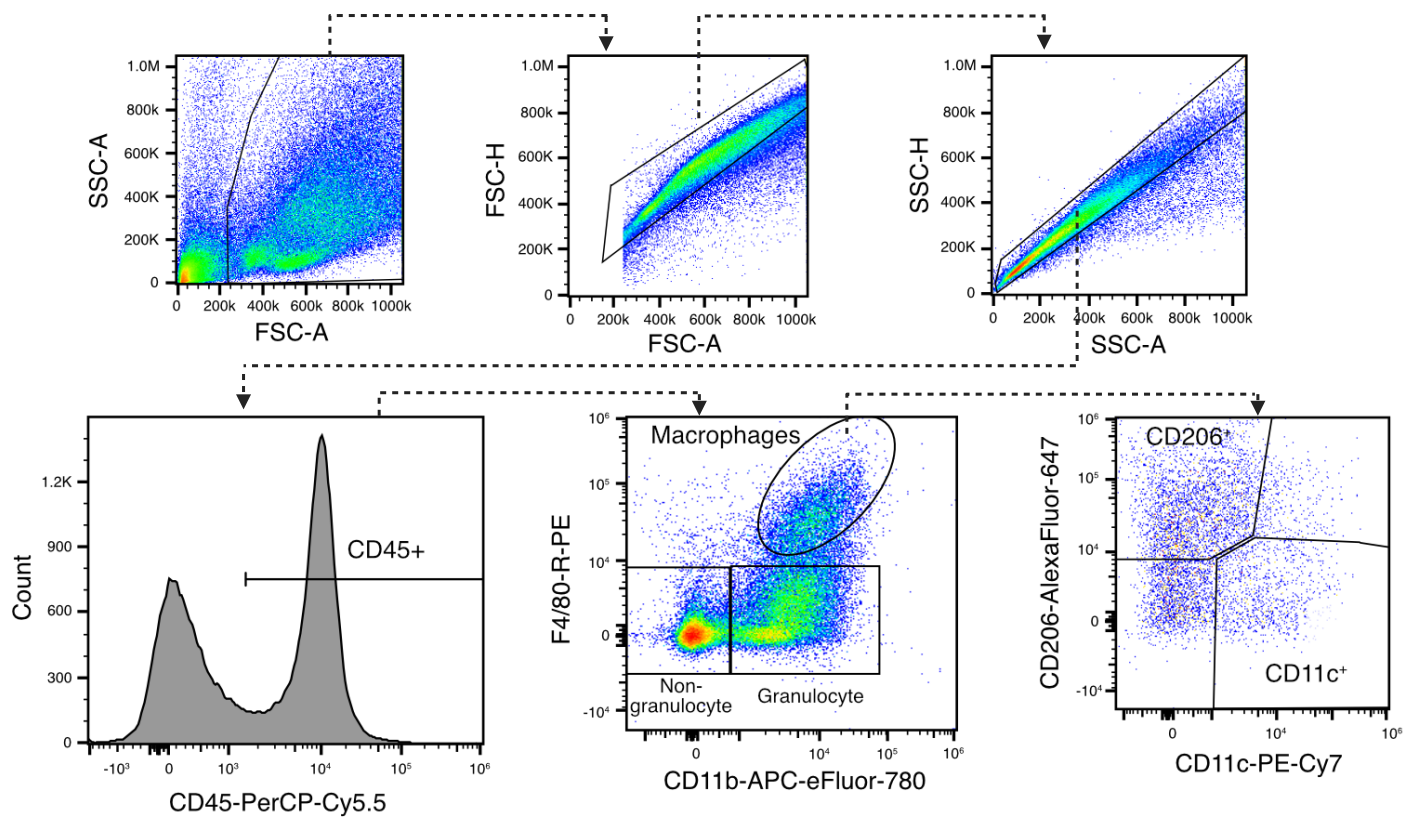

B

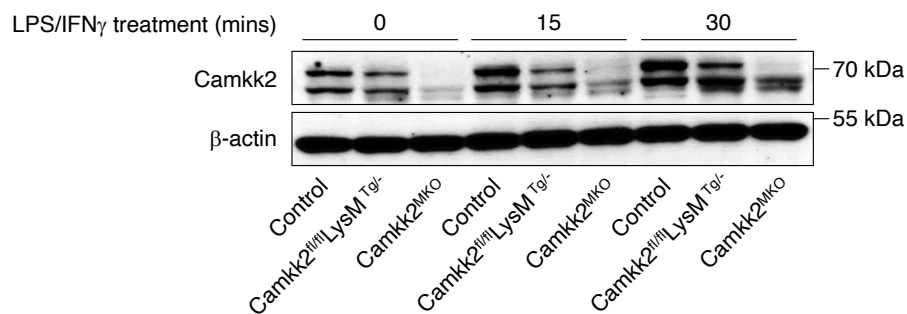

Supplement: Figure S2 — Representative gating strategy to identify macrophages subsets in eWAT (A). Debris, doublets and non-viable cells were first excluded, after which total tissue leukocytes were identified as CD45+. The macrophage pool was identified as F4/80+CD11b+, from which CD11c+ and CD206+ macrophages were identified. (B) Immunoblot of time course of Camkk2 expression in BMDMs treated with lipopolysaccharide (LPS) and interferon-γ (IFNγ) from control and Camkk2MKO mice. [file mmc2.pdf]
